# Impaired bone healing upon neutrophil-specific adrenoreceptor beta 2 knockout in non-osteoporotic and osteoporotic mice

**DOI:** 10.1038/s41536-026-00481-y

**Published:** 2026-05-28

**Authors:** Sandra Dieterich, Nico Gläser, Christoph Kölbl, Dorothea Gebauer, Jana Bleher, Oliver Küppers, Verena Fischer, Anita Ignatius, Melanie Haffner-Luntzer

**Affiliations:** Institute of Orthopaedic Research and Biomechanics, University Medical Center Ulm, Ulm, Germany

**Keywords:** Diseases, Immunology, Medical research, Physiology

## Abstract

After a bone fracture, osteoporotic mice show delayed healing associated with elevated systemic inflammation and increased neutrophil numbers in the early fracture hematoma. Because short-term treatment with the beta-adrenoreceptor-blocker propranolol reduced neutrophil recruitment, we hypothesized that deletion of β_2_-adrenoreceptor (Adrb2) signaling in neutrophils would normalize neutrophil recruitment and accelerate bone healing in osteoporotic mice. A conditional Adrb2 knockout in Ly6G⁺ neutrophils was generated using Ly6G-Cre Adrb2-flox mice. Bone and immune phenotypes were analyzed via µCT and histology under non-fracture conditions and during bone healing in ovariectomized postmenopausal mice. Both non-osteoporotic and osteoporotic female Ly6G-Adrb2-KO mice showed impaired bone healing vs. controls, while only minor bone alterations were observed under non-fracture conditions. Pathway analysis of isolated neutrophils, characterized by RNA-sequencing, suggested reduced neutrophil activation and disturbed neutrophil/mast cell interactions upon Ly6G-Adrb2-KO. Summarily, our data demonstrate that Adrb2 signaling is important for neutrophil recruitment and seems to be critical for proper bone healing.

## Introduction

It is well established that the skeletal and the immune system are closely interconnected in many aspects, a concept that has given rise to the interdisciplinary field of osteoimmunology. Immune cells such as T cells, macrophages, and neutrophil granulocytes critically influence bone metabolism through the secretion of mediators such as receptor activator of nuclear factor kappa-B ligand (RANKL), tumor necrosis factor-alpha (TNF-α), or interleukin-6 (IL-6), thereby modulating both osteoclastic bone resorption and osteoblastic bone formation^1,2^. One clinical example of a disrupted osteoimmunological balance is postmenopausal osteoporosis, which is characterized by an imbalance in bone metabolism, with increased activity of bone-resorbing osteoclasts relative to bone-forming osteoblasts. This imbalance is driven not only by the direct positive effects of estrogen on bone formation and its inhibitory effects on bone resorption, but also by the fact that estrogen deficiency after menopause induces a chronic low-grade inflammatory state^3,4^, which further contributes to impaired bone homeostasis. As a result, osteoporotic bone exhibits an altered microarchitecture that increases fracture risk^5^.

Each year, more than 3.5 million new osteoporotic fractures occur in Europe, and 10–15% of patients die within the first year after suffering from an osteoporotic hip fracture^6,7^. In addition to the increased fracture risk, osteoporotic bone displays a reduced capacity for fracture healing^8^, as demonstrated in numerous animal studies using ovariectomized, estrogen-deficient rodents. These animals exhibit a mechanically weaker fracture callus with increased osteoclast numbers^9–12^, reduced expression of cartilage markers^13^, and impaired expression of angiogenic factors^14^. Furthermore, we previously demonstrated that ovariectomized mice exhibit elevated systemic levels of proinflammatory cytokines and increased numbers of neutrophil granulocytes in the fracture hematoma 3 days after osteotomy, which was associated with delayed fracture healing^15^. However, the mechanisms underlying the enhanced recruitment and/or activity of neutrophils in osteoporosis and osteoporotic bone healing remain largely unclear.

Emerging evidence suggests a role for adrenergic signaling in this process, as neutrophil granulocytes are strongly influenced by catecholamines and adrenergic stimulation^16,17^. Catecholamines play a pivotal physiological role, as they are released in response to external and internal stressors via activation of the hypothalamic–pituitary–adrenal (HPA) axis. By binding to distinct adrenergic receptor subtypes expressed across multiple organs and tissues, catecholamines regulate essential physiological functions, including cardiovascular activity, pupillary diameter, and autonomic control of gastrointestinal processes^18,19^. Bone is also subject to sympathetic regulation, which influences both bone formation and turnover. For example, treatment of stressed male mice with the non-selective β-adrenergic receptor antagonist propranolol immediately before fracture significantly reduced the number of neutrophil granulocytes in the fracture hematoma and improved fracture healing^20^. In this context, it is important to note that neutrophil granulocytes, similar to osteoblasts and osteoclasts^21^, express the β_2_-adrenoreceptor (Adrb2)^22,23^. This receptor is thought to play a key role in adrenergic regulation of bone metabolism, as previous studies demonstrated that both global^24,25^ and osteoblast-specific^26^ Adrb2 knockout (KO) led to increased bone volume in mice. However, the role of Adrb2 signaling in immune cells in the context of bone metabolism and fracture healing remains unknown.

Therefore, the aim of this study was to elucidate the role of Adrb2 in Ly6G⁺ neutrophil granulocytes during bone healing in both non-osteoporotic and osteoporotic mice. We hypothesized that catecholamine release following osteotomy activates Adrb2 signaling in neutrophils, thereby promoting their enhanced recruitment to the fracture hematoma and contributing to impaired bone regeneration in osteoporotic bone. To test this hypothesis, mice with a neutrophil-specific deletion of Adrb2 were generated. Bone metabolism upon KO was analyzed in both sexes, while bone healing was investigated in female mice after ovariectomy, a model of postmenopausal osteoporosis.

## Results

**Fig. 1:**
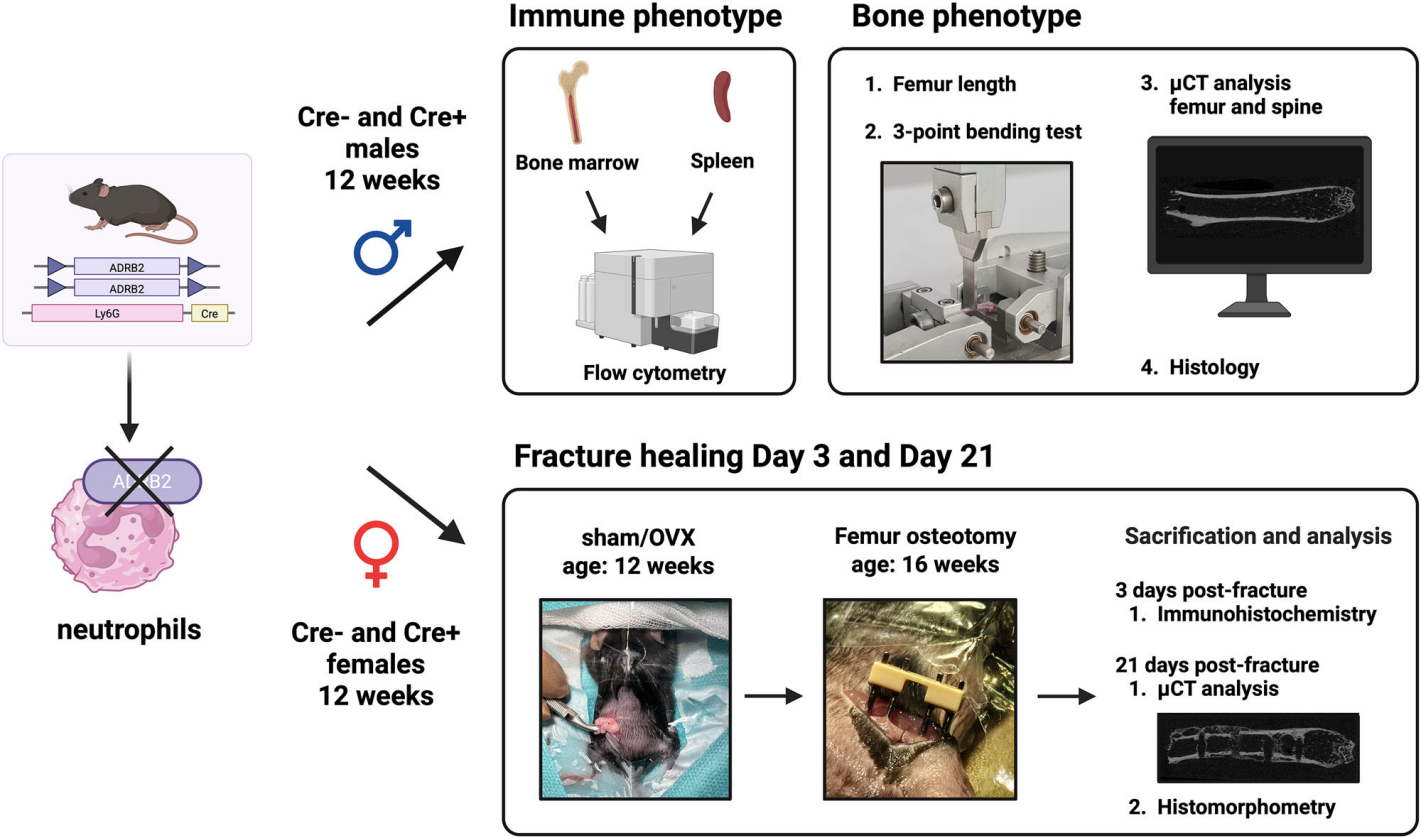
Experimental setup of the phentoyping procedure using male mice (♂) and the bone healing study using female mice (♀). 12-week-old mice with (Cre^+^) and without (Cre^−^) a specific knockout in the β_2_-adrenergic receptor on neutrophils were created using the Cre/loxP-system. Firstly, the immune and the bone phenotype of male mice were analyzed. After that, bone healing of female mice that underwent either ovariectomy (OVX) or sham-surgery was evaluated on day 3 and day 21 after a standardized femur osteotomy at the age of 16 weeks. Created in BioRender. Ohmayer, W. (2026) https://BioRender.com/9k7ic8s.

**Fig. 2:**
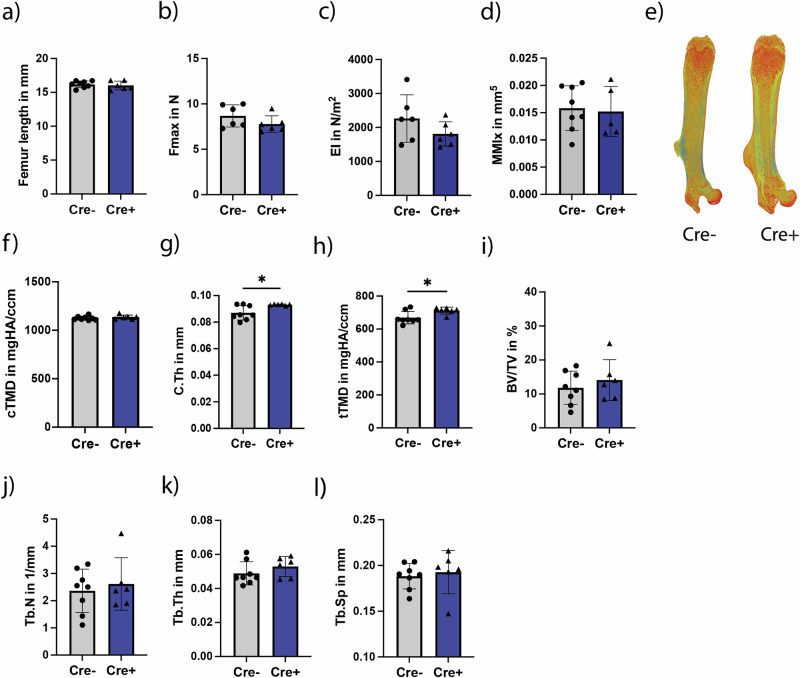
Bone phenotyping of 12-week-old male Ly6G-Cre/Adrb2-flox KO mice. Following parameters were evaluated: **a** Femur length in mm. **b** *F*_max_ in N. **c** Bending stiffness (EI in N/m^2^). **d** Cortical moment of inertia (MMIx) in mm^5^. **e** Representative 3D-reconstruction images of Cre^−^ and Cre^+^ male mice. **f** Cortical tissue mineral density (TMD) in mgHA/ccm. **g** Cortical thickness (C.Th). **h** Trabecular TMD in mgHA/ccm. **i** Femoral trabecular BV/TV in %. **j** Femoral trabecular number in 1/mm. **k** Femoral trabecular thickness in mm. **l** Femoral trabecular separation in mm. Statistical significance was determined by an unpaired *t*-test (comparison Cre^−^ vs. Cre^+^). Dots and gray bars represent Cre^−^ mice, triangles and blue bars represent Cre^+^ mice. Each dot and triangle represents one animal. *P < 0.05, **P < 0.01, ***P < 0.001, ****P < 0.0001 (*N* = 6–8; males).

First, the immune and bone phenotype of 12-week-old male Ly6G-Cre/Adrb2-flox mice was assessed to analyze differences under non-fracture conditions upon KO (Fig. 1). Immune cell analysis showed no differences in CD19^+^, CD11b^+^/Ly6G^+^, CD11b^+^/F4/80^+^, CD3^+^/CD4^+^, and CD3^+^/CD8^+^ cells in spleen and bone marrow in *Adrb2* KO mice compared to control mice (Table 1). Bone phenotyping revealed that the bone parameters femur length (Fig. 2a), maximal load to failure (Fig. 2b), bending stiffness (Fig. 2c), cortical moment of inertia (MMIx)(Fig. 2d), cortical tissue mineral density (TMD) (Fig. 2f), trabecular bone volume fraction (BV/TV) (Fig. 2i), trabecular number (Tb.N) (Fig. 2j), trabecular thickness (Tb.Th)(Fig. 2k), trabecular separation (Tb.Sp) (Fig. 2l), number of osteoblasts per bone perimeter (Supplementary Fig. 1a), and number of osteoclasts per bone perimeter (Supplementary Fig. 1b) were unaffected. In addition, assessment of the lumbar spine showed no differences in BV/TV, Tb.N, Tb.Th and Tb.Sp (Supplementary Fig. 2). Cre^+^ male mice showed a slightly increased trabecular tissue mineral density (tTMD) in the femur (Fig. 2h) and increased cortical thickness (C.Th) (Fig. 2g) with increased numbers of osteocytes in the cortex (Supplementary Fig. 1c) compared to Cre^−^ littermates. Figure 2e shows 3D-reconstruction images of representative mice of each group.

**Table 1:** Flow cytometry antibodies

Antibody	Label	Product	Company	Dilution
CD11b (rat anti-mouse)	Alexa Fluor® 700	56-0112-82	Thermo Fisher Scientific, Inc.	1:200
CD19 (rat anti-mouse)	PE	12-0193-81	Thermo Fisher Scientific, Inc.	1:200
CD3e (rat anti-mouse)	PE-Cyanine7	25-0031-82	Thermo Fisher Scientific, Inc.	1:200
CD4 (rat anti-mouse)	APC-e-Fluor® 780	47-0042-82	Thermo Fisher Scientific, Inc.	1:200
CD8a (rat anti-mouse)	APC	17-0081-81	Thermo Fisher Scientific, Inc.	1:200
F4/80 (rat anti-mouse)	FITC	11-4701-82	Thermo Fisher Scientific, Inc.	1:50
Ly6G (rat anti-mouse)	V450	11-4801-82	BD Bioscience	1:200
IgG Isotype (Armenian hamster)	PE-Cyanine7	25-4888-81	Thermo Fisher Scientific, Inc.	1:200
IgG2a K Isotype (rat)	Alexa Fluor® 700	IC006N	R&D Systems, Inc.	1:50
IgG2a K Isotype (rat)	APC	17-4321-81	Thermo Fisher Scientific, Inc.	1:200
IgG2a K Isotype (rat)	PE	12-4321-81	Thermo Fisher Scientific, Inc.	1:200
IgG2a K Isotype (rat)	FITC	11-4321-42	Thermo Fisher Scientific, Inc.	1:100
IgG2b K Isotype (rat)	APC-eFluor® 780	47-4031-82	Thermo Fisher Scientific, Inc.	1:200
IgG2a K Isotype (rat)	V450	560377	BD Bioscience	1:200

**Fig. 3:**
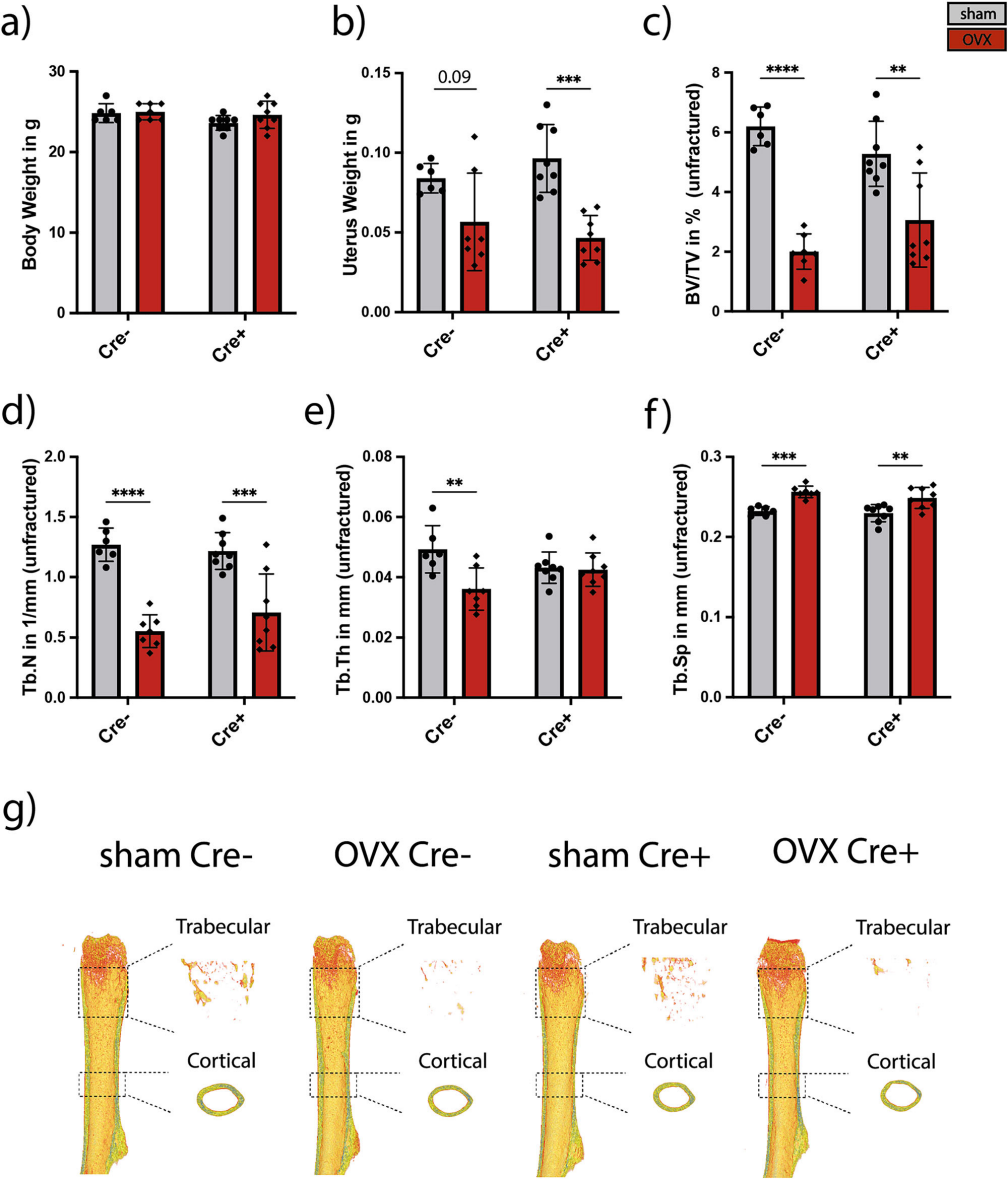
Analysis of body weight, uterus weight and trabecular bone parameters of the contralateral unfractured femur 21 days after osteotomy. **a** Mouse body weight in g. **b** Uterus weight in g. **c** BV/T V in %. **d** Tb.N in 1/mm. **e** Tb.Th in mm. **f** Tb.Sp in mm. **g** 3D-reconstruction images of the cortical and trabecular bone. Statistical significance was determined by Two-way ANOVA. Dots and gray bars represent the sham group, rhombs and red bars represent the OVX group. Each dot and triangle represents one animal. *P < 0.05, **P < 0.01, ***P < 0.001, ****P < 0.0001 (*N* = 5–8; females).

To investigate the role of Adrb2-signaling on neutrophils during bone healing in non-osteoporotic and osteoporotic bone, 12-week-old female mice underwent OVX to induce an osteoporotic bone phenotype driven by estrogen depletion. The mice were randomly distributed to the different groups and did not display differences in body weight 21 days after osteotomy (Fig. 3a). Successful OVX was confirmed by a reduced uterus weight in OVX mice (Fig. 3b). Furthermore, µCT analysis of the contralateral unfractured femur revealed a significant reduction of BV/TV (Fig. 3c) and Tb.N (Fig. 3d), while Tb.Sp (Fig. 3f) was increased in both Cre^−^ and Cre^+^ OVX mice compared to the respective sham group (Fig. 3g). This indicates that OVX induced an osteoporotic bone phenotype irrespective of the genotype. In addition, we observed that both sham and OVX Cre^+^ female mice display no bone phenotype compared to the respective Cre^−^ group, as demonstrated by the absence of differences in BV/TV, Tb.N, Tb.Th, and Tb.Sp of the unfractured femur (Fig. 3c–g).

**Fig. 4:**
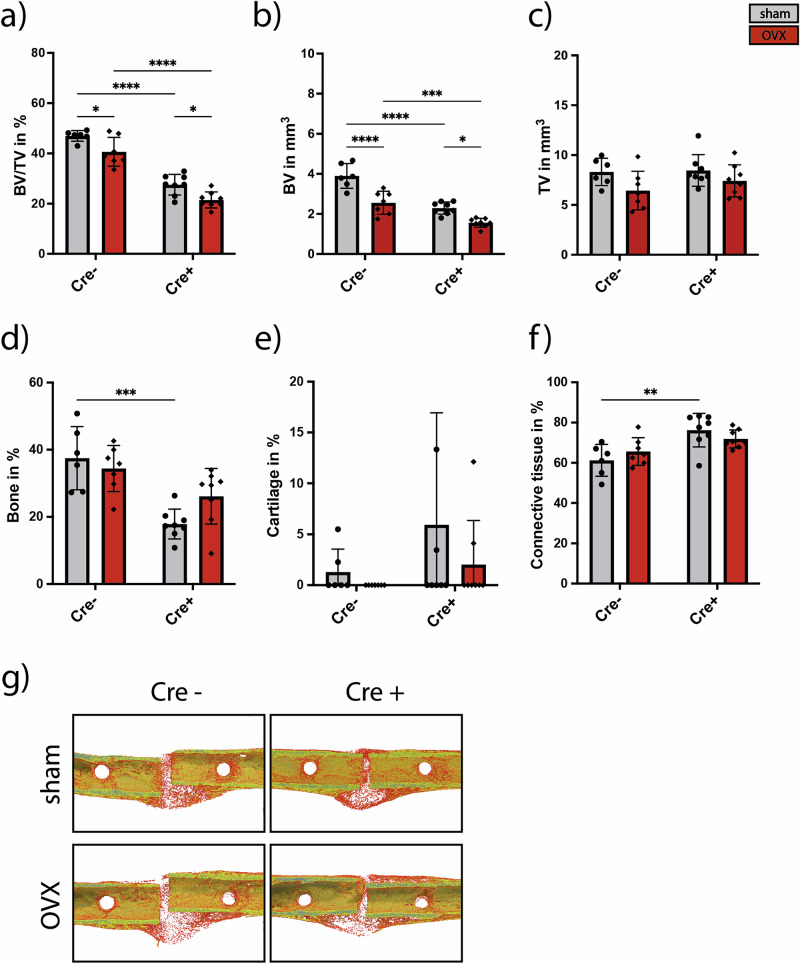
µCT analysis and histomorphometry at day 21 after osteotomy. **a** Callus bone volume per tissue volume (BV/TV) in % as determined by µCT. **b** Callus bone volume (BV in mm^3^). **c** Callus tissue volume (TV in mm^3^). 2D Histomorphometry analysis; Percentage of bone (**d**), cartilage (**e**) and connective tissue (**f**) in the newly formed callus gap. **g** 3D-reconstruction images of the fracture callus from representative mice of each group. Yellow = mature bone and red = newly formed bone. Statistical significance was determined by Two-way ANOVA. Dots and gray bars represent the sham group, rhombs and red bars represent the OVX group. Each dot and triangle represents one animal. *P < 0.05, **P < 0.01, ***P < 0.001, ****P < 0.0001 (*N* = 6–8; females).

Bone-healing outcome 21 days after surgery was investigated using µCT analysis and histomorphometry. 3D µCT analysis depicted significantly reduced BV/TV and bone volume (BV) in OVX mice compared to sham mice, while tissue volume (TV) was unaffected (Fig. 4a–c), irrespective of genotype, indicating delayed healing due to OVX in both groups. More strikingly, BV/TV and BV were significantly decreased in both Cre^+^ groups compared to their respective Cre^-^ group (Fig. 4a, b), indicating delayed healing due to the Adrb2-KO. Representative 3D reconstruction images of the fracture calli are depicted in Fig. 4g. In addition, two-dimensional histomorphometry revealed a significant decrease in bone and increased connective tissue content in the fracture gap of sham Cre^+^ mice compared to sham Cre^−^ mice, supporting the µCT data (Fig. 4d–f).

**Fig. 5:**
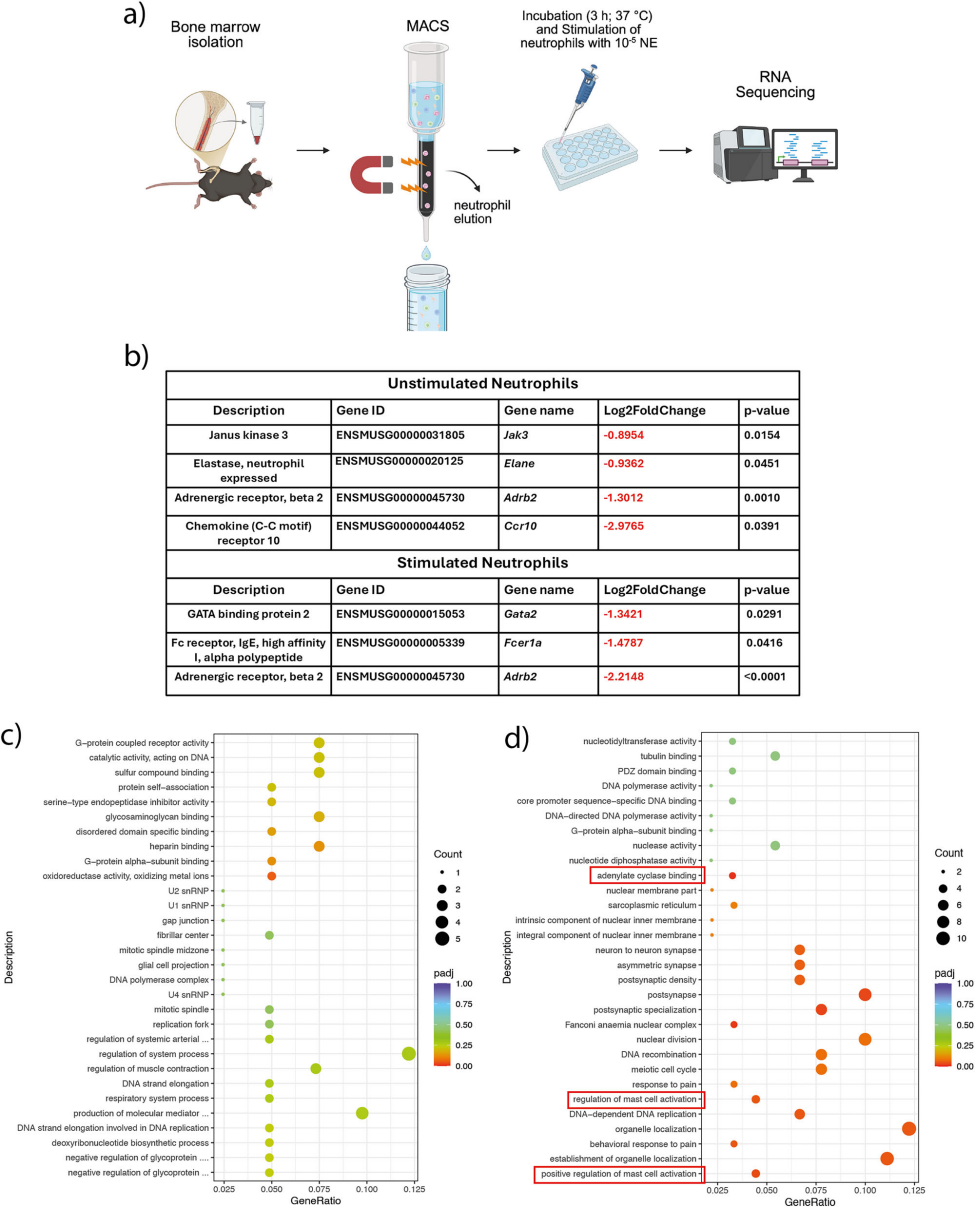
Neutrophil isolation via magnetic activated cell sorting (MACS), stimulation with norepinephrine and RNA-sequencing analysis. **a** Experimental design. Created in BioRender. Ohmayer, W. (2026) https://BioRender.com/2omqke5. **b** Differentially expressed genes in the comparison of unstimulated and stimulated Cre^−^ and Cre^+^ neutrophils. **c** GO enrichment analysis from significantly downregulated genes in unstimulated Cre^−^ vs. Cre^+^ neutrophils. **d** GO enrichment analysis from significantly downregulated genes in stimulated Cre^−^ vs. Cre^+^ neutrophils. **c** and **d** The abscissa is the ratio of the number of differential genes linked with the GO pathway to the total number of differential genes. The ordinate is the GO Pathway. The size of a point represents the number of genes annotated to a specific GO pathway. The color scale represents the significance level of the enrichment (*N* = 3; males).

To further elucidate the role of *Adrb2* deletion in neutrophils and investigate underlying molecular mechanisms of the delayed healing in Cre^+^ mice, RNA-Sequencing of isolated neutrophils from the bone marrow of Cre^−^ and Cre^+^ male mice was conducted. Half of the cells were stimulated with 10^–5^ M norepinephrine (Fig. 5a) to induce the Adrb2 pathway. Unstimulated neutrophils revealed 48 upregulated and 86 downregulated genes upon Adrb2 KO, while an additional noradrenaline stimulation resulted in 148 upregulated and 135 downregulated genes (Supplementary Fig. 3). A list of all significantly regulated genes for unstimulated and stimulated neutrophils is provided in the supplements (Supplemental Data 1 and 2). Within those, the most interesting genes were Janus kinase 3 *(Jak3)*, Elastase *(Elane)*, the Adrenergic receptor beta 2 *(Adrb2)*, Chemokine (C–C motif) receptor 10 *(Ccr10),* Gata2 *(Gata2)*, and the Fc receptor *(Fcer1a)* (Fig. 5b). *Jak3*, *Elane*, *Adrb2* and *Ccr10* were significantly downregulated in unstimulated Cre^+^ neutrophils (Fig. 5b), while *Gata2*, *Fcer1a and Adrb2* showed significantly downregulated gene expressions in stimulated Cre^+^ neutrophils (Fig. 5b). In addition, *Adrb2* expression in whole bone lysates was significantly decreased upon KO which supports the RNA sequencing data and confirms the effectiveness of our KO model (Supplementary Fig. 4a). GO enrichment analysis of unstimulated Cre^+^ vs. Cre^−^ neutrophils indicated alteration in neutrophil cellular functions upon KO (Fig. 5c), while GO enrichment analysis of stimulated Cre^+^ vs. Cre^−^ neutrophils display several mast cell-related terms (Fig. 5d). The significantly downregulated pathways “regulation of mast cell activation” and “positive regulation of mast cell activation” in stimulated Cre^+^ vs. Cre^−^ neutrophils could imply a potentially affected neutrophil/mast cell interaction upon neutrophil-specific Adrb2 KO (Fig. 5d). Supplementary Fig. 5 shows the GO enrichment analysis from significantly upregulated genes in unstimulated (a) and stimulated (b) Cre^+^ vs. Cre^−^ neutrophils, which mainly reveals a regulation of peptidases upon KO.

**Fig. 6:**
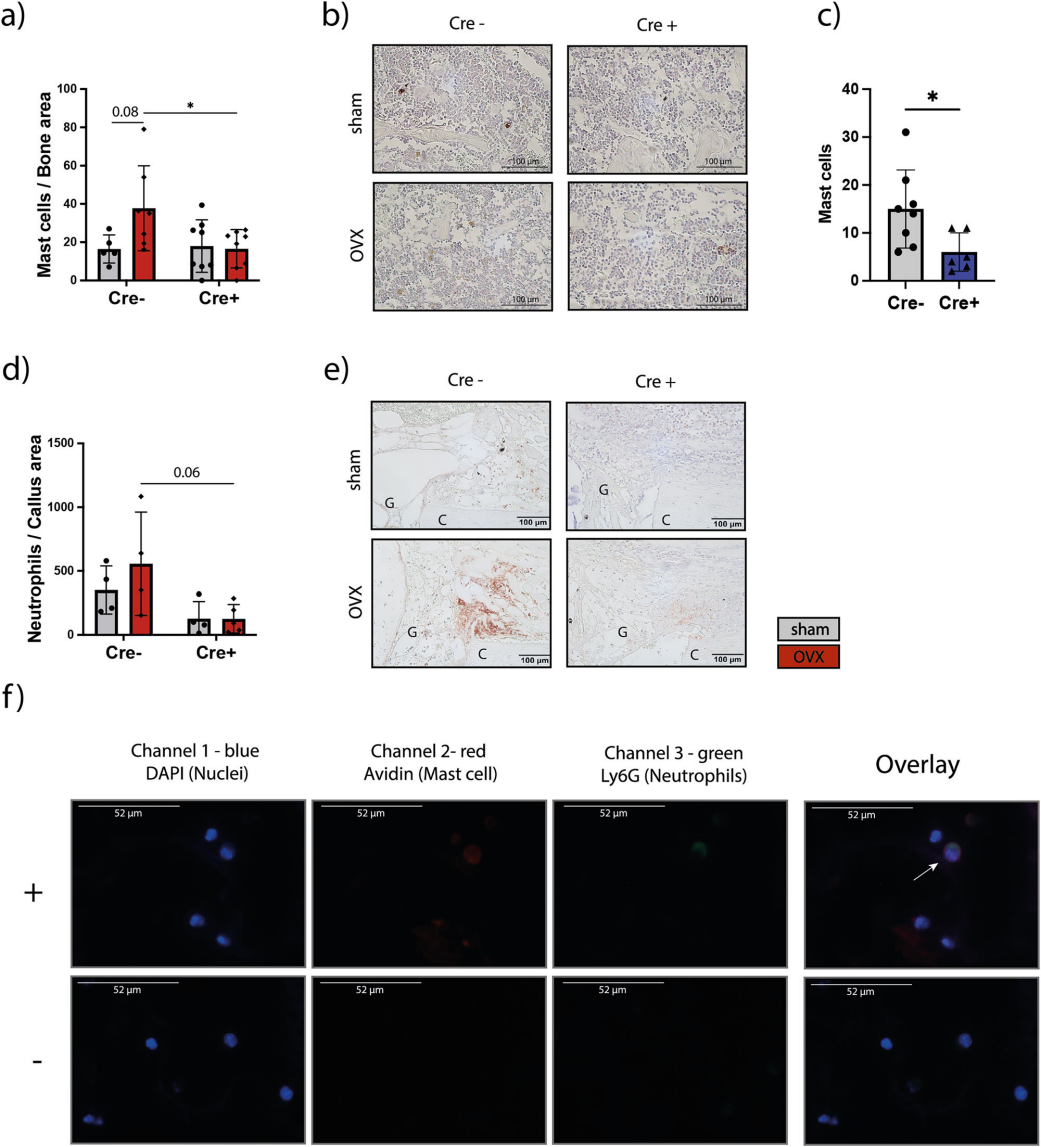
Immunohistochemistry stainings of neutrophils and mast cells in the fracture callus 3- and 21-days post-osteotomy. **a** Mast cell ratio (mast cell numbers/bone area) in the fracture gap of female mice 21 days post-osteotomy. **b** Representative images of mast cells stained in the fracture callus 21 days post-osteotomy. **c** Mast cell numbers in the intact femur of male mice; gray = Cre^−^; blue = Cre^+^. **d** Neutrophils per callus area in the fracture callus of female mice 3 days post-osteotomy**. e** Representative images of the neutrophil (Ly6G) staining in the fracture callus of female mice 3 days post-osteotomy. G = Gap, C = Cortex. **f** + = Doublestaining of neutrophils (Ly6G; green) and mast cells (Avidin; red) showing MIT formation^27^ (highlighted with an arrow) in the fracture gap of female mice 3 days post-osteotomy. Nuclei are stained in blue with DAPI. − = negative control with species-specific, non-targeting immunoglobulins. Statistical significance was determined by unpaired *t*-test (comparison Cre^−^ vs. Cre^+^)(**c**) and two-way ANOVA (**a** and **d**). Dots and gray bars represent the sham group (**a** and **d**) and Cre^−^ mice (**c**), rhombs and red bars represent the OVX group (**a** and **d**), triangles and the blue bar represent Cre^+^ mice (**c**). Each dot and triangle represents one animal. *P < 0.05, **P < 0.01, ***P < 0.001, ****P < 0.0001 (*N* = 4–8).

Because of the finding that *Adrb2* deletion on neutrophils might affect neutrophil and mast cell recruitment/activation, we analyzed mast cell numbers in the fracture callus of sham and OVX female mice 21 days post-osteotomy (Fig. 6a, b). The analysis revealed no significant differences between sham and OVX mice of both genotypes (Fig. 6a, b). However, female Cre^+^ OVX mice showed a significantly reduced percentage of mast cells compared to Cre^−^ OVX mice (Fig. 6a). This is in line with significantly reduced mast cell numbers in the intact femur of Cre^+^ male mice (Fig. 6c). To further investigate the effect of Adrb2 deletion on neutrophils, we performed a neutrophil (Ly6G) and macrophage (F4/80) staining in the fracture callus 3 days post-osteotomy, where we observed a strong reduction of neutrophil recruitment in Cre^+^ mice compared to Cre^−^ littermates (Fig. 6d, e) while macrophage presence was unaffected (Supplementary Fig. 6). In addition, we performed a double staining of neutrophils (Ly6G) and mast cells (Avidin) to investigate the recently described mechanism of MIT formation^27^, where mast cells engulf neutrophils, during bone healing. Indeed, we could show neutrophils trapped intracellularly in mast cells in the early fracture hematoma (Fig. 6f). Taken together, these data indicate that neutrophil-specific deletion of the Adrb2 may impair neutrophil recruitment and reduce mast cell numbers by an unknown mechanism.

## Discussion

The aim of this study was to investigate the effect of a neutrophil-specific *Adrb2* deletion on bone homeostasis and bone healing in non-osteoporotic and osteoporotic bone. Our hypothesis was that the deletion of the Adrb2 would accelerate bone healing, specifically under osteoporotic conditions, due to reduced neutrophil recruitment. In contrast to this initial hypothesis, we could show that Adrb2-KO on neutrophils had a moderate sex-specific positive effect on intact bone, while bone healing was significantly delayed in both non-osteoporotic and osteoporotic conditions.

A conditional KO of the *Adrb2* on Ly6G-expressing cells was generated using the Cre/loxP system, which results in reduced Adrb2 expression on neutrophils, confirmed by our RNASeq data. First, the immune and bone phenotype of Cre^−^ and Cre^+^ male littermates was assessed. Male mice were chosen to address 3R principles, as we used their female littermates for the bone-healing study. Nevertheless, the bone phenotype of female Cre^−^ and Cre^+^ mice was also analyzed in this study using the contralateral intact left femur. Flow cytometric analysis of male mice revealed no significant differences in immune cell populations following Adrb2 knockout (KO). In contrast, a moderate bone phenotype was observed, characterized by increased cortical thickness, elevated osteocyte numbers, and higher trabecular TMD. Although these alterations were modest, they suggest that neutrophil-specific deletion of Adrb2 may exert subtle positive effects on bone homeostasis under non-fracture conditions in male mice. One possible explanation is that altered neutrophil signaling indirectly affects osteocyte function or bone remodeling dynamics through changes in local paracrine communication, inflammatory mediator release, or coupling signals between bone-resorbing and bone-forming cells. However, as the underlying mechanisms were not directly investigated in our study, further targeted analyses will be required to fully elucidate the pathways responsible for these bone-specific alterations in Cre^+^ male mice. In contrast, female Cre^+^ mice with a femur fracture did not display a bone phenotype upon neutrophil-specific Adrb2 KO in their contralateral non-fractured femur. It has been shown previously that a fracture also affects the rest of the skeleton, which might explain the differences between male and female bone phenotype in our study^28^.

Next, we investigated bone repair, beginning with the induction of postmenopausal osteoporosis at 12 weeks of age by ovariectomy, followed by a standardized femoral osteotomy stabilized with an external fixator at 16 weeks of age. Analysis of uterine weight confirmed the expected uterine atrophy in both Cre^−^ and Cre⁺ ovariectomized (OVX) mice. Consistently, µCT analysis of the unfractured femur demonstrated the characteristic decrease in bone volume fraction (BV/TV) in OVX mice, independent of genotype. These findings indicate that neutrophil-specific deletion of Adrb2 does not protect mice from the development of osteoporosis. Although osteoporosis has been associated with an increased sympathetic tone^29^, our data suggest that adrenergic signaling in neutrophils does not contribute to osteoporosis development. In contrast, in osteoblasts, β_2_-adrenergic signaling is known to play a critical role in the pathogenesis of osteoporosis^26^.

µCT analysis of the fracture calli at 21 days post-fracture revealed significantly reduced bone volume fraction and bone volume in OVX mice compared with sham-operated mice of both genotypes, indicating impaired bone healing in Cre^−^ and Cre⁺ osteoporotic mice. These findings suggest that neutrophil-specific Adrb2 knockout does not rescue delayed bone healing in osteoporotic mice. In contrast, Cre⁺ sham and OVX mice exhibited significantly poorer bone healing than their respective Cre^−^ controls, indicating that neutrophil-specific deletion of Adrb2 impairs bone healing under both physiological and osteoporotic conditions. These findings are contradictory to our initial hypothesis and indicate that Adrb2 signaling specifically in neutrophils is of great importance for successful bone healing. In line with our findings, a recent study by Jahn et al. has demonstrated that treatment with propranolol, a nonspecific beta-adrenergic blocker, impaired fracture healing in mice^30^, although previous studies observed opposite results^20,29,31^. The different results of these studies may depend on different study designs and may indicate a time and/or concentration-dependent influence of drug treatment.

To further elucidate our findings, we performed a more detailed characterization of neutrophils using an in vitro culture approach followed by RNA sequencing. Neutrophils were isolated from the bone marrow of Cre^−^ and Cre⁺ mice and either stimulated with noradrenaline or cultured in medium alone as a control. RNA sequencing identified several differentially expressed genes in both unstimulated and noradrenaline-stimulated Cre⁺ neutrophils compared with Cre^−^ neutrophils, including *Jak3*, *Elane*, *Adrb2*, *Ccr10*, *Gata2*, and *Fcer1a*. The significant downregulation of *Adrb2* in unstimulated neutrophils of Cre+ mice and qPCR of whole bone lysate indicate the effectiveness of the knockout. *Jak3* encodes a non-receptor tyrosine kinase that plays a critical role in neutrophil activation in response to interleukin-8 (IL-8) during inflammatory reactions^32^. Moreover, *Jak3* has been shown to be essential for neutrophil chemotaxis^32,33^. This indicates that neutrophil chemotaxis might be affected upon *Adrb*2 KO. Ccr10 is known as an important receptor for the immune response and plays a role in the recruitment of immune cells, but less is known about its function in neutrophils^34^. In addition, *Elane* encodes for neutrophil-expressed elastase, which is known as an important factor in promoting inflammation, e.g., in various lung diseases^35^. Other downregulated candidate genes were *Fcer1a* and *Gata2*. *Fcer1a* encodes for a high-affinity IgE receptor playing a central role in allergic diseases and is mainly expressed on mast cells but in a smaller manner also on neutrophils^35,36^. *Gata2* is an important transcription factor regulating *Fcer1a* expression, which means that the downregulation of *Gata2* results in a downregulation of *Fcer1a* expression^37^. GO enrichment analysis of noradrenaline-stimulated neutrophils further indicates the possibility of a disturbed neutrophil/mast cell interaction since the GO term “(positive) regulation of mast cell activation” was significantly downregulated upon Ly6G-Adrb2-KO. Elastase could be involved in this regulation, as it is known to recruit mast cells. In general, mast cells derive from hematopoietic stem cells of the bone marrow and are mainly known for their function in IgE-mediated allergies and tissue repair^38,39^. But they are also associated with age-related and postmenopausal osteoporosis as indicated by increased mast cell numbers and the ability to regulate osteoclastic activity^40–42^. Using mast cell deficient mice, we demonstrated previously that mast cells activate osteoclastic bone resorption in postmenopausal osteoporosis and are critically involved in osteoporotic bone healing^43–45^. Furthermore, severely injured mice showed increased mast cell numbers in the fracture hematoma, while mast cell-depleted mice were protected against severe injury-induced impairment of fracture healing^46^. In summary, these data demonstrate that increased mast cell numbers under conditions of hyperinflammation can be detrimental to bone regeneration. Based on these observations, we decided to further investigate mast cell populations in our mouse model. Immunohistochemical staining was performed to quantify mast cells in the intact femur of male mice and in the fracture callus of female mice. Our data revealed that Cre⁺ male mice exhibited significantly reduced mast cell numbers in the intact bone. In the fracture callus, this reduction of mast cells was also observed in Cre⁺ osteoporotic mice, which displayed significantly fewer mast cells in the fracture callus compared to Cre^−^ osteoporotic controls. Despite the reduction of mast cell numbers, Cre⁺ mice still exhibited delayed bone healing, which is challenging to reconcile since in previous fracture healing studies in models of hyperinflammation (e.g., osteoporosis), mast cell deficiency resulted in improved fracture healing. Currently, we have no proven explanation for our findings. One possible explanation could be that the neutrophil–mast cell crosstalk is disrupted in this model and that a balanced neutrophil–mast cell crosstalk is important for bone healing. Previous studies have shown that neutrophils recruit mast cells to sites of inflammation through the secretion of chemokines such as CXCL1 and CXCL12^47^. An altered chemokine expression or signaling in Adrb2-deficient neutrophils could therefore impair mast cell recruitment and function, leading to a dysregulated inflammatory response and ultimately compromising bone healing. To further understand this neutrophil/mast cell interaction, we stained neutrophils in the fracture callus 3 days post-fracture, where we observed a drastically reduced neutrophil recruitment in Cre^+^ mice. Interestingly, macrophage recruitment was unaffected upon KO. This implies that Ly6G-Adrb2 KO mice exhibit disturbed neutrophil activation and an impaired neutrophil/mast cell crosstalk, resulting in reduced numbers of both cell types in the fracture callus. As previously described by Kovtun et al., neutrophils play a crucial role in bone fracture healing^48^. Treatment with a Ly6G antibody led to impaired bone regeneration, highlighting the importance of undisturbed neutrophil recruitment and function in the early inflammatory phase of fracture healing^48^. This imbalance of neutrophils and mast cells in our mouse model may explain the poor bone-healing outcome observed in Cre^+^ mice, as a finely tuned activation and interaction has been shown to be essential for successful bone repair.

Despite their central role in innate immunity-mediating antimicrobial defense through phagocytosis, degranulation, and the formation of neutrophil extracellular traps (NETs)—neutrophils also appear to exert important immunomodulatory functions during tissue repair^49^. In this context, Mihlan et al. recently described a process termed mast cell-induced neutrophil trapping (MIT), in which degranulating mast cells secrete leukotriene B4 to attract neutrophils and retain them within mast cells. The trapped neutrophils subsequently undergo cell death, while their components are retained by mast cells, thereby enhancing mast cell metabolic fitness and functional capacity^27^. Notably, we were able to detect MIT formation in the fracture callus at 3 days post-osteotomy using immunofluorescent double staining for neutrophils and mast cells. Nevertheless, this observation alone does not explain the impaired bone healing observed in Ly6G-Adrb2 KO mice. One plausible explanation could be that reduced neutrophil activation and recruitment in these mice limit the initiation and regulation of early inflammatory signaling cascades required for effective mast cell activation, angiogenesis, and subsequent tissue remodeling. Consequently, insufficient early inflammatory signaling may lead to a failure to properly transition from the inflammatory to the reparative phase of fracture healing.

In conclusion, our findings highlight the importance of a tightly regulated neutrophil–mast cell interaction during bone regeneration and suggest that both insufficient and excessive immune cell activity can be detrimental to bone healing. Adrb2 signaling on neutrophils seems to be important in that context. Further studies are clearly required to dissect the molecular mechanisms underlying this bidirectional crosstalk and to determine how its dysregulation contributes to impaired bone repair in osteoporotic conditions.

Limitations of our study are that our Ly6G-Cre model affects not only neutrophils but also other myeloid-derived cells expressing Ly6G, e.g., some types of monocytes or myeloid-derived suppressor cells. Furthermore, investigating earlier time points after osteotomy, neutrophil functions and other involved immune cells, e.g., T- and B-cells, to further characterize the neutrophil/mast cell crosstalk during bone healing would be needed for future studies. Another limitation of this study is the use of the Ly6G-Cre driver, which represents a knock-in into the endogenous Ly6g locus and may potentially affect Ly6G expression; however, given that Ly6G is primarily considered a neutrophil marker rather than a key functional regulator, a major impact on our findings appears unlikely. However, using Cre-positive wt/wt mice as another control group would have been needed to further prove that. Furthermore, we did not include non-fractured female mice in the study due to 3R reasons to reduce the number of animals.

In summary, our data show that Adrb2-signaling on neutrophils is not relevant for the development of postmenopausal osteoporosis, while it seems to be critical for bone healing in both non-osteoporotic and osteoporotic mice. This could be due to an impaired activation of neutrophils and a disturbed interaction of neutrophils and mast cells upon Ly6G-Adrb2-KO, which needs to be further investigated.

## Methods

### Animal housing and transgenic mouse model

All animal experiments were reported in compliance with the European Guidelines for Animal Research on the Protection of Animals and the ARRIVE guidelines and were approved by the local animal welfare authority (Regierungspräsidium Tübingen, No. 1612). Mice were housed in groups of up to five per cage under a 12-h light/12-h dark cycle with ad libitum access to food and water. The study utilized transgenic mice with a C57BL/6J background carrying a neutrophil-specific KO of the Adrb2 under control of the Cre/loxP system (Ly6G-Cre⁺ Adrb2^flox/flox^). This mouse line was established by breeding Ly6G-Cre mice^50^ with Adrb2flox mice kindly donated by Gerard Karsenty^51^. Cre-negative littermates (Ly6G-Cre^−^ Adrb2^flox/flox^) were included as controls.

### Experimental design and surgical procedures

In the first part of the study, male Cre^+^ and Cre^−^ mice were euthanized by isoflurane overdose and terminal intracardiac blood withdrawal at the age of 12 weeks to analyze their bone and immune phenotype via µCT analysis, histology and flow cytometry (Fig. 1). Male littermate mice were used in accordance with the 3R principle of reduction, as the females were required for the osteotomy surgeries and the males could be included in the phenotyping study without generating additional breeding pairs. To assess the bone phenotype in female mice, the unfractured contralateral femur was analyzed via µCT.

The second part of the study focused on bone healing in female non-osteoporotic and osteoporotic mice. Twelve-week-old female mice were subjected to either a sham operation or bilateral ovariectomy (OVX) to induce postmenopausal osteoporosis as previously described^4,15,52^. Anesthesia was performed starting with 5–6% isoflurane (Forene, Abbott) and maintained with 2% isoflurane (Forene, Abbott) with an oxygen flow rate of 0.7 mL/min. To ensure adequate analgesia, all mice received tramadol (25 mg/L, Tramal, Gruenenthal GmbH) in their drinking water from one day pre- until 3 days postoperatively. After surgery, the normal housing diet (ssniff R/M-H, V1535-300, Ssniff GmbH) was changed to a phytoestrogen-low diet (ssniff R/M-H, V1554-300, Ssniff GmbH). Four weeks post-surgery, a standardized, unilateral transverse osteotomy of the right femur was performed, which was stabilized using an external fixator as previously described^53^. Mice were euthanized by isoflurane overdose on days 3 and 21 post-osteotomy for subsequent analyses, including µCT analysis, histology, and IHC staining (Fig. 1).

### Biomechanical testing

To assess the mechanical properties of the bones, biomechanical testing was conducted on intact femurs from 12-week-old male mice. A destructive three-point bending test was performed following a previously established protocol^53^. Each bone was subjected to a load of up to 10 N using a materials testing machine (Zwick Roell, Ulm, Germany), while load and deflection were continuously recorded. For each bone, the maximal load until failure (*F*_max_) was calculated, and the flexural rigidity was determined from the slope of the linear region of the load–deflection curve.

### μCT analysis

Fractured and unfractured femurs were fixed in 4% paraformaldehyde for 48 h prior to micro-computed tomography (μCT) scanning. Imaging was performed using a Skyscan 1172 device (Skyscan, Aartselaar, Belgium) at a peak voltage of 50 kV and a current of 200 μA to assess bone content and mineralization. The isotropic voxel resolution was set to 8 μm. Three-dimensional analysis was conducted using computed tomography analysis (CTAn) and CT volume (CTVol) software (Bruker) in accordance with ASBMR guidelines^54^. Analyzing the fractured bones, the volume of interest (VOI) was defined as the entire periosteal callus region located between the two inner pinholes of the fixator. Unfractured bones were analyzed in two VOIs: VOI 1 encompassed a region extending 360 µm from the proximal end of the growth plate to 280 µm proximal to the distal end of the bone. VOI 2 covered the area from the proximal end of the trochanter tertius to 80 µm proximal to the distal end. Tissue mineral density was quantified using calibration phantoms containing defined hydroxyapatite (HA) concentrations of 250 mg HA/cm³ and 750 mg HA/cm³. The threshold for mineralized tissue was set at 394 mg HA/cm^3^ for trabecular bone and 642 mg HA/cm³ for cortical bone and fracture callus analysis.

### Histomorphometry

Decalcified histological analysis of fractured and unfractured femurs was performed as previously described^55^. Sections of 4 μm thickness were stained with Safranin O/Fast Green to assess tissue composition and with tartrate-resistant alkaline phosphatase (TRAP) to identify osteoclasts. In the fractured bones, the proportions of bone, cartilage, and fibrous tissue within the fracture callus at day 21 post-osteotomy were quantified in Safranin O-stained sections using image analysis software (Leica MMAF 1.4.0 Imaging System; Leica, Wetzlar, Germany). The region of interest (ROI) of the fractured samples was defined as the entire fracture callus between the two inner pinholes of the fixator.

In addition, osteoblasts and osteoclasts were quantified in sections of fractured and unfractured femora at 20x magnification. In unfractured samples, the ROI (480 × 350 µm) for quantifying osteoblasts and osteoclasts was defined as an area located 960 µm proximal to the distal growth plate and positioned between both cortices in a trabecular-rich area, excluding cortical bone, as previously described^18^. Osteocytes were quantified at the same longitudinal level but within the cortical bone. All cells fully embedded within the cortical matrix were classified as osteocytes. In fractured samples, the same area was defined in the periosteal callus near the fracture gap. Osteoblasts were identified and counted in Safranin O-stained sections based on their characteristic morphology as cubic-shaped cells lining the bone surface. Osteoclasts were analyzed in TRAP-stained sections and identified by their positive TRAP staining, distinctive multinucleated morphology, size, and localization on the bone surface.

### Immunohistochemistry

Longitudinal sections of 4 μm thickness were prepared for immunohistochemical staining. Detection of Mcpt5 was performed using the primary antibody rabbit anti-mouse MC Protease 5 (1:100; orb11030, Biorbyt, Cambridge, UK), which was incubated overnight at 4 °C. The secondary antibody goat anti-rabbit IgG-biotin (1:200; B2770, Life Technologies, Carlsbad, CA, USA) was applied at room temperature (RT) for 1 h. Detection of Ly6G was performed using the primary antibody rat anti-mouse Ly6G (1:300; 127632, BioLegend, San Diego, USA), which was incubated overnight at 4 °C. The secondary antibody goat anti-rat IgG-biotin (1:200; #31830, Invitrogen, Carlsbad, CA, USA) was applied at RT for 1 h. Detection of F4/80 was performed using the primary antibody rat anti-mouse F4/80 (1:500; #MCA497GA, Bio-Rad Laboratories, Feldkirchen, Germany), which was incubated overnight at 4 °C. The secondary antibody goat anti-rat IgG-biotin (1:200; #31830, Invitrogen, Carlsbad, CA, USA) was applied at RT for 1 h. For all single stainings, horseradish peroxidase (HRP)-conjugated streptavidin (PK-6100, VECTASTAIN Elite ABC-HRP Kit, Peroxidase, Vector Laboratories, Burlingame, UK) was used for signal detection according to the manufacturer’s instructions. NovaRED (SK-4800, Vector NovaRED Substrate Kit, Peroxidase (HRP), Vector Laboratories) served as the chromogen, and sections were counterstained with hematoxylin (1:2000; 2C-306, Waldeck, Münster, Germany). Species-specific, non-targeting immunoglobulins were used as isotype controls. A total of 4–8 mice per group were analyzed. Neutrophils (Ly6G^+^), mast cells (Mcpt5^+^) and macrophages (F4/80+) were quantified within a defined volume of interest (480 × 350 µm) in an area of the fracture callus with the maximum amount of cells at 20x magnification. Identification was based on characteristic staining, morphology, and cell size.

### Immunofluorescent staining Ly6G-Avidin

A double staining of neutrophils and mast cells in the fracture callus 3 days after osteotomy was performed following the subsequent protocol. Detection of Ly6G was performed using the primary antibody rat anti-mouse Ly6G (1:300; #127632, BioLegend, San Diego, USA), which was incubated overnight at 4 °C. The secondary antibody, rabbit anti-rat IgG (H + L) Alexa Fluor 488 (A-21210; Thermo Fisher, Waltham, MA, USA) and Avidin Texas Red (A820, Invitrogen, Carlsbad, CA, USA), which stains mast cell granules, were applied at RT for 1 h. Nuclei were stained with DAPI (D9542; Sigma-Aldrich, St. Louis, MO, USA) for 10 min at RT. Species-specific, non-targeting immunoglobulins were used as isotype controls. Stained slides were screened for possible mast cell intracellular trap (MIT) formation.

### Flow cytometry

Flow cytometry was performed to characterize immune cell populations in the bone marrow and spleen of Cre^−^ and Cre^+^ 12-week-old male mice. The bone marrow was flushed out of the left femur using 10 mL phosphate-buffered saline (PBS). The spleen was harvested and passed through a 70-µm cell strainer (Corning Inc., Durham, NC, USA). Cells from both the spleen and bone marrow underwent erythrolysis to remove red blood cells. For immunophenotyping, macrophages (F4/80^+^), neutrophils (Ly-6G^+^), inflammatory monocytes (CD11b^+^), B-lymphocytes (CD19^+^), T-lymphocytes (CD3^+^), cytotoxic T-lymphocytes (CD3^+^, CD8^+^), and T-helper lymphocytes (CD3^+^, CD4^+^) were identified using the antibodies listed in Table 2. Isotype-matched immunoglobulin antibodies (Table 1) served as negative controls. The isolated cells were incubated with the respective antibodies for 30 min on ice. Dead-cell discrimination was performed using 7-aminoactinomycin D (7-AAD, Sigma, Steinheim, Germany). Flow cytometric analysis was conducted on a FACSLyric flow cytometer (BD Bioscience), and data were analyzed using FlowJo software v10 (FlowJo LLC, Ashland, OR).

**Table 2:** Immune cell phenotyping using flow cytometry

	Spleen					Bone marrow				
Cell type/Genotype	CD19^+^	CD11b^+^/Ly6G^+^	CD11b^+^/F4/80^+^	CD3^+^/CD4^+^	CD3^+^/CD8^+^	CD19^+^	CD11b^+^/Ly6G^+^	CD11b^+^/F4/80^+^	CD3^+^/CD4^+^	CD3^+^/CD8^+^
Cre^−^	50.9 ± 4.6	2.6 ± 0.8	6.5 ± 1.4	16.8 ± 3.0	20.7 ± 5.3	17.6 ± 2.7	35.4 ± 8.1	12.1 ± 3.2	0.9 ± 0.2	1.1 ± 0.2
Cre^+^	50.1 ± 3.5	3.4 ± 1.4	4.8 ± 1.4	15.4 ± 2.5	18.5 ± 3.9	14.2 ± 3.7	32.1 ± 6.5	11.4 ± 3.0	1.0 ± 0.4	1.1 ± 0.4

Percentage of CD19^+^, CD11b^+^/Ly6G^+^, CD11b^+^/F4/80^+^, CD3^+^/CD4^+^, and CD3^+^/CD8^+^ cells. Statistical significance was determined by an unpaired *t*-test (comparison Cre^−^ vs. Cre^+^) (*N* = 6; males).

### Magnetic-activated cell sorting (MACS) of bone marrow neutrophils

Following bone marrow flushing from long bones of intact mice, red blood cells were lysed using 5 mL erythrocyte lysis buffer (5 min, 37 °C). The suspension was subsequently centrifuged at 1500 rpm for 5 min at 4 °C. After removing the supernatant, the cell pellet was resuspended in 200 µL MACS buffer. The suspension was filtered through a 30-μm cell strainer with an additional 400 µL MACS buffer, and the total cell number was determined. 5 × 10⁷ cells were centrifuged at 300×*g* for 10 min at 4 °C, and the supernatant was discarded. For negative selection of neutrophils, 50 µL of a neutrophil-specific biotin antibody cocktail was added to the cell pellet, mixed, and incubated for 10 min at 2–8 °C. This antibody cocktail binds to all non-neutrophilic cells in the suspension, leaving neutrophils untouched. After washing with 5–10 mL MACS buffer, cells were centrifuged (300×*g*, 10 min), the supernatant was aspirated, and the pellet was resuspended in 400 µL MACS buffer. Next, 100 µL of anti-biotin MicroBeads were added, mixed, and incubated for 15 min at 2–8 °C, allowing them to bind to the biotinylated antibodies. After an additional wash with 5–10 mL MACS buffer and a final centrifugation (300×*g*, 10 min), the supernatant was discarded, and the pellet was resuspended in 500 µL MACS buffer. For magnetic cell separation, the cell suspension was applied to an LS-MACS column pre-rinsed with 3 mL MACS buffer and placed in a QuadroMACS separator. This step allowed the separation of neutrophils from other labeled cells, which were retained by the column, while neutrophils passed through and were collected for further analysis.

### RNAseq analysis of isolated neutrophils

Neutrophils were isolated from the long bones of three Cre^−^ and three Cre^+^ male mice by MACS as described above. For each mouse, the isolated neutrophils were split into two wells. One well was stimulated for 3 h with 10^−5^ M norepinephrine at 37 °C, while the other well served as an unstimulated control. For RNA isolation using the RNeasy Mini Kit (74106; Qiagen, Hilden, Germany), neutrophils were resuspended in 400 µL RPMI medium supplemented with 10% (v/v) FCS after two rounds of centrifugation (300×*g*, 10 min) and washing in PBS. Cell numbers were determined prior to stimulation. After a 3 h stimulation period, cells were centrifuged (300×*g*, 10 min), washed with 200 µL PBS, and centrifuged again (300×*g*, 10 min). The supernatant was removed, and the final cell pellet of each subgroup was resuspended in 350 µL RLT buffer for RNA isolation. Lysates were stored at −80 °C until RNA sequencing. RNAseq was performed by the Novogene Corporation (Munich, Germany). The workflow began with sample quality control (Sample QC) to ensure that the samples met the RNAseq criteria, including RNA quantity and RNA integrity number (RIN). Following this, the appropriate RNA library was prepared and tested for quality (Library QC). The RNA library was constructed through polyadenylated (polyA) capture or ribosomal RNA (rRNA) removal, followed by reverse transcription to cDNA. Sequencing was conducted using Illumina PE150 technology (Illumina, San Diego, CA, USA) with a paired-end 150-bp sequencing strategy. The resulting data were subjected to quality control (Data QC). Gene expression differences with a *p*-value of <0.05 between the groups were considered differentially regulated. Bioinformatic analyses were carried out on the differentially regulated genes, including Gene Ontology (GO) term enrichment analysis and Kyoto Encyclopedia of Genes and Genomes (KEGG) pathway analysis. ClusterProfiler software was used for enrichment analysis. GO http://www.geneontology.org/ is a widely used bioinformatics classification system that categorizes gene properties across species into three main branches: cellular component, molecular function, and biological process. GO terms with an adjusted probability (padj) < 0.05 were considered significantly enriched. KEGG is a curated database containing genomic, biological pathway, and disease information. Pathway enrichment analysis identifies significantly enriched metabolic or signaling pathways associated with differentially expressed genes, comparing them to the entire genomic background. KEGG pathways with padj < 0.05 were considered significantly enriched.

### RNA isolation, cDNA synthesis and qPCR of whole bone lysates

Total RNA was isolated from the whole femur of 4 Cre^−^ and 4 Cre^+^ male mice using the PureLink RNA Mini Kit (Invitrogen, Cat. 12183018A, Carlsbad, CA, USA) according to the manufacturer’s instructions. cDNA synthesis was performed with the Omniscript reverse transcriptase kit (Qiagen, Hilden, Germany, Cat. 205113). Quantitative real-time PCR (qRT-PCR) was conducted using the CFX Opus 96 Real-Time PCR System (Bio-Rad, Feldkirchen, Germany). The gene expression level of *Adrb2* was analyzed using the delta-delta CT method and normalized to the housekeeping gene beta-2-mikroglobulin (*B2m*).

### Statistics

A priori power calculations were performed for the primary outcomes to ensure adequate statistical power to detect biologically relevant differences between groups. For this calculation, data from a previous study have been used^15^. Statistical analyses were conducted using an unpaired *t*-test and Two-way analysis of variance (ANOVA) followed by Šidák’s post hoc test. A significance level of *p* < 0.05 was applied. Data in figures and tables are depicted as bars with mean ± standard deviation. Each experimental group consisted of 3–8 animals, with exact group sizes provided in the figure legends.

## Supplementary information

Supplementary.

Supplementary Data 1.

Supplementary Data 2.

## Data Availability

All data are available in the main text or the supplementary materials. The raw datasets used and/or analyzed during the current study are available as supplementary files. In addition, the lists of all differentially expressed genes were uploaded to Zenodo, 10.5281/zenodo.18229993.
