# Supplementary material for: Impaired bone healing upon neutrophil-specific adrenoreceptor beta 2 knockout in non-osteoporotic and osteoporotic mice
Source: NPJ Regen Med. 2026 May 28;11:24. doi: 10.1038/s41536-026-00481-y (PMC13219604; doi:10.1038/s41536-026-00481-y)
Supplement: Supplementary file 1 — Supplementary. [file 41536_2026_481_MOESM1_ESM.pdf]

Supplemental

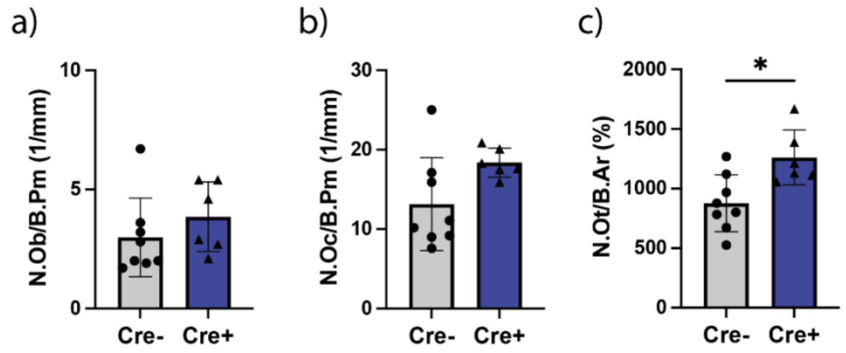

Supp.Fig. 1: Femoral osteoblast, osteoclast and osteocyte analysis. **a)** Number of osteoblasts per bone perimeter in 1/mm. **b)** Number of osteoclasts per bone perimeter in 1/mm. **c)** Number of osteocytes per bone area in %. Statistical significance was determined by unpaired t-test (comparison Cre$^-$ vs. Cre$^+$). Dots and grey bars represent Cre$^-$ mice, triangles and blue bars represent Cre$^+$ mice. Each dot and triangle represent one animal. $^*P < 0.05$, $^{**}P < 0.01$, $^{***}P < 0.001$, $^{****}P < 0.0001$. (*N*=6-8; males)

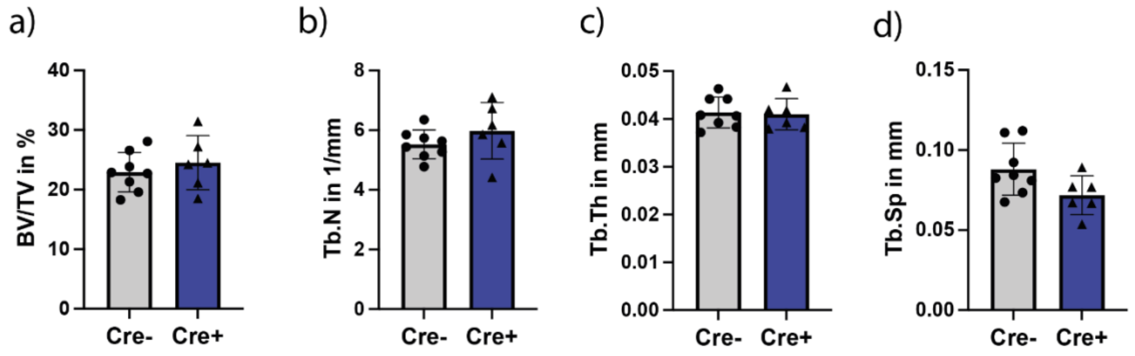

Supp.Fig. 2: Spine phenotyping of 12 week old male Ly6G-Cre/Adrb2-flox KO mice using µCT analysis. a) Spine BV/TV in %. **b)** Spine trabecular number in 1/mm. **c)** Spine trabecular thickness in mm. **d)** Spine trabecular separation in mm. Statistical significance was determined by unpaired t-test (comparison Cre$^-$ vs. Cre$^+$). Dots and grey bars represent Cre$^-$ mice, triangles and blue bars represent Cre$^+$ mice. $^*P < 0.05$, $^{**}P < 0.01$, $^{***}P < 0.001$, $^{****}P < 0.0001$. (*N*=6-8; males)

a) b)

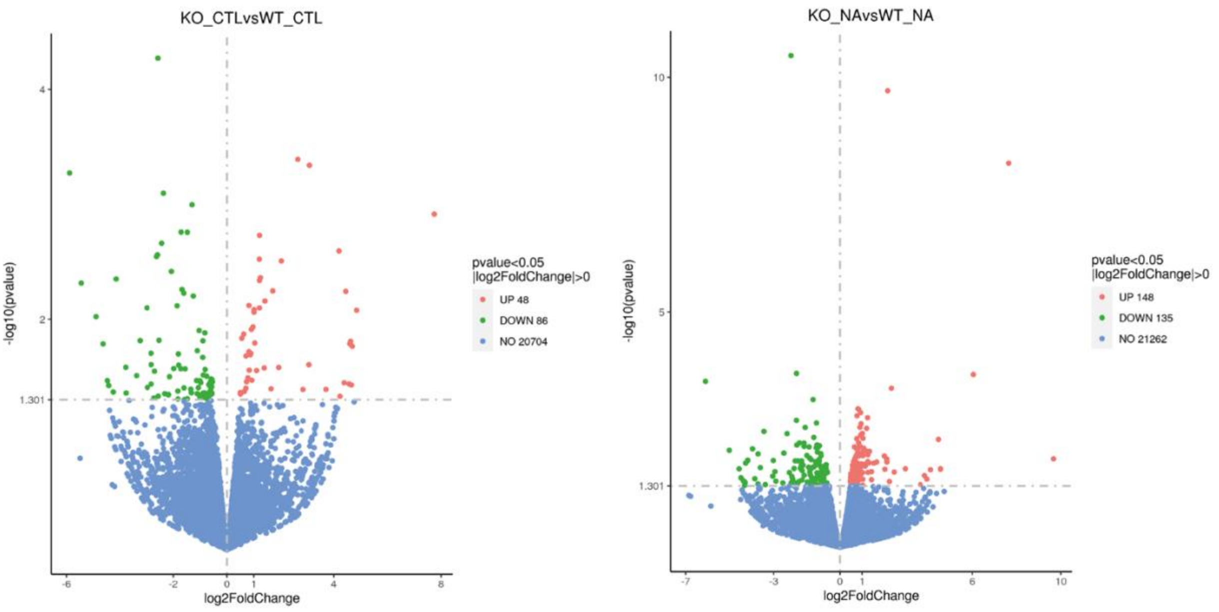

Supp.Fig. 3: Overview of all up- and downregulated genes in unstimulated and stimulated Cre+ neutrophils. **a)** Comparison of Cre- and Cre+ unstimulated neutrophils. **b)** Comparison of Cre- and Cre+ stimulated neutrophils. Red dots represent upregulated genes, green dots represent downregulated genes, blue dots represent genes without significant different regulation. (*N*=3; males)

a)

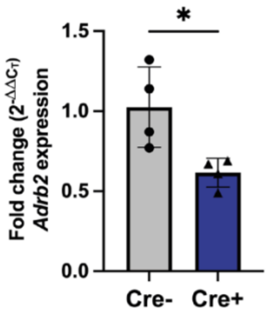

Supp. Fig. 4: Adrb2 qPCR of whole bone lysates. a) Fold change in *Adrb2* expression of whole bone lysates of 4 Cre^- and 4 Cre^+ male mice. Significantly downregulated *Adrb2* expression in Cre^+ mice. Statistical significance was determined by unpaired t-test. Dots and grey bars represent Cre^- mice, triangles and blue bars represent Cre^+ mice. Each dot and triangle represent one animal. *P < 0.05. (*N*=4; males)

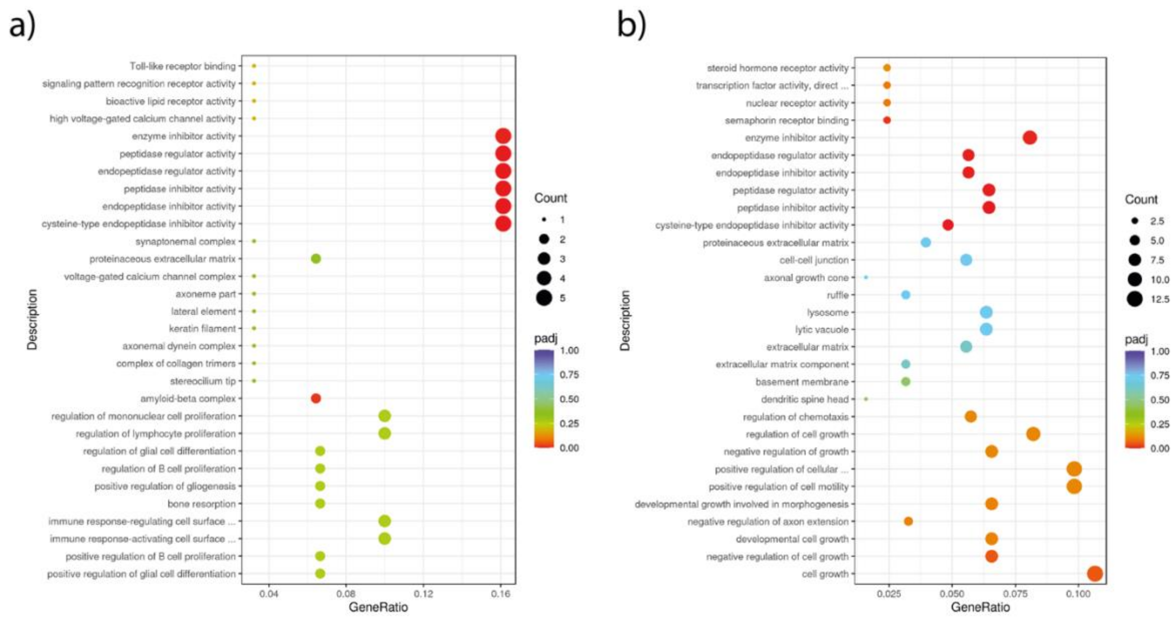

Supp.Fig. 5: GO enrichment analysis from significantly upregulated genes in non-stimulated and stimulated Cre^{+} neutrophils vs. Cre^{-} neutrophils. a) Comparison of Cre- and Cre+ unstimulated neutrophils. **b)** Comparison of Cre- and Cre+ stimulated neutrophils. Red dots represent upregulated genes, green dots represent downregulated genes, blue dots represent genes without significant different regulation. (*N*=3; males)

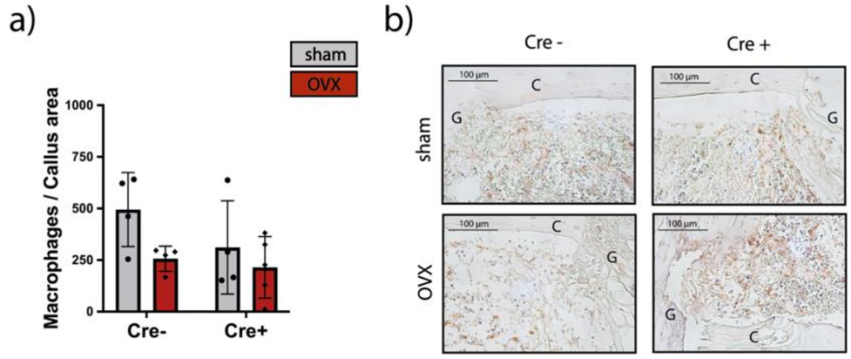

Supp. Fig. 6: Macrophage (F4/80) staining in the fracture callus 3 days post-osteotomy. a) Quantitative analysis macrophages in the fracture gap. **b)** Representative images of the macrophage staining (F4/80). C = Cortex; G = Gap. Statistical significance was determined by Two-way ANOVA. Dots and grey bars represent Cre^{-} mice, triangles and blue bars represent Cre^{+} mice. Each dot and triangle represent one animal. *P < 0.05, **P < 0.01, ***P < 0.001, ****P < 0.0001. (*N*=4-5; females)

Additional data as excel files

Supplemental Data 1: Excel list of all differentially regulated genes in unstimulated Cre^+ neutrophils compared to unstimulated Cre^- neutrophils.

Supplemental Data 2: Excel list of all differentially regulated genes in stimulated Cre^+ neutrophils compared to stimulated Cre^- neutrophils.
